# Out-of-pocket expenditure and catastrophic health spending on maternal care in public and private health centres in India: a comparative study of pre and post national health mission period

**DOI:** 10.1186/s13561-017-0167-1

**Published:** 2017-09-18

**Authors:** Sanjay K. Mohanty, Anshul Kastor

**Affiliations:** 1 Department of Fertility Studies, International Institute for Population Sciences (IIPS), Govandi Station Road, Deonar, Mumbai, 400088 India; 2 Research Scholar, International Institute for Population Sciences, Mumbai, India

**Keywords:** National Health Mission, National Rural Health Mission, Maternal care, Delivery care, Catastrophic health spending, Out-of-pocket expenditure, India

## Abstract

**Background:**

The National Health Mission (NHM), one of the largest publicly funded maternal health programs worldwide was initiated in 2005 to reduce maternal, neo-natal and infant mortality and out-of-pocket expenditure (OOPE) on maternal care in India. Though evidence suggests improvement in maternal and child health, little is known on the change in OOPE and catastrophic health spending (CHS) since the launch of NHM.

**Aim:**

The aim of this paper is to provide a comprehensive estimate of OOPE and CHS on maternal care by public and private health providers in pre and post NHM periods.

**Data and method:**

The unit data from the 60th and 71st rounds of National Sample Survey (NSS) is used in the analyses. Descriptive statistics is used to understand the differentials in OOPE and CHS. The CHS is estimated based on capacity to pay, derived from household consumption expenditure, the subsistence expenditure (based on state specific poverty line) and household OOPE on maternal care. Data of both rounds are pooled to understand the impact of NHM on OOPE and CHS. The log-linear regression model and the logit regression models adjusted for state fixed effect, clustering and socio-economic and demographic correlates are used in the analyses.

**Results:**

Women availing themselves of ante natal, natal and post natal care (all three maternal care services) from public health centres have increased from 11% in 2004 to 31% by 2014 while that from private health centres had increased from 12% to 20% during the same period. The mean OOPE on all three maternal care services from public health centres was US$60 in pre-NHM and US$86 in post-NHM periods while that from private health center was US$170 and US$300 during the same period. Controlling for socioeconomic and demographic correlates, the OOPE on delivery care from public health center had not shown any significant increase in post NHM period. The OOPE on delivery care in private health center had increased by 5.6 times compared to that from public health centers in pre NHM period. Economic well-being of the households and educational attainment of women is positively and significantly associated with OOPE, linking OOPE and ability to pay. The extent of CHS on all three maternal care from public health centers had declined from 56% in pre NHM period to 29% in post NHM period while that from private health centres had declined from 56% to 47% during the same period. The odds of incurring CHS on institutional delivery in public health centers (OR .03, 95% CI 0.02, 06) and maternal care (OR 0.06, 95% CI 0.04, 0.07) suggest decline in CHS in the post NHM period. Women delivering in private health centres, residing in rural areas and poor households are more likely to face CHS on maternal care.

**Conclusion:**

NHM has been successful in increasing maternal care and reducing the catastrophic health spending in public health centers. Regulating private health centres and continuing cash incentive under NHM is recommended.

## Background

Reduction of maternal mortality, neonatal and under-five mortality and financial risk protection are three key health related targets of sustainable development goals (SDGs) [1]. Achieving health related SDGs required significant investment in maternal and child health to protect households from high out-of-pocket expenditure (OOPE) and catastrophic health spending (CHS). High OOPE is positively associated with CHS and reduced access to health services, increases untreated morbidity, reduce consumption of goods and services, and lead to long-term impoverishment [2]. The level of CHS varies across countries, among socio-economic groups and by nature and type of health services. Cross country studies suggest that households with low educational attainment, lower economic status, without health insurance and residing in rural areas are more likely to incur CHS [3–6]. The OOPE and CHS are high for maternal services in many developing countries including India [7, 8].

Estimates of OOPE and CHS on health care are gaining increasing research and programmatic attention. A growing number of studies from developing countries suggest that the health care payment has increased the poverty level and affect the poor most [9–11]. Globally, OOPE studies on maternal care addressed socioeconomic and demographic differentials and estimated the incidence and correlates of CHS. The general findings suggest higher OOPE for caesarean delivery, complicated delivery, deliveries in private health centres and for higher socioeconomic groups [12–16]. The global progress in improvement of maternal and child health and reduction of CHS is contingent on India’s success on these indicators.

### Maternal and child health in pre and post NHM periods in India

India with one-sixth of the world’s population, federal nature of governance and sustained economic growth, is undergoing health transition. The healthcare system in the country is characterized by the presence of both public (central, state and local government) and private health care providers, varying delivery structures and multiple systems of medicine [17]. While health is a state subject (within the state government) the central government helps in policy making, planning, guiding, evaluating and providing the funding to implement the national program. The utilization of health services from private health care providers is generally linked to the ability to pay and quality of care.

A decade ago, the state of maternal and child health in India was extremely poor. The maternal mortality ratio (MMR) and the infant mortality rate (IMR) were high. About three-fifths of the mothers were delivering without any medical assistance and women perceived cost as a major barrier to the utilization of maternal care. As a policy response, the Government of India launched the National Rural Health Mission (NRHM) in 2005 to improve the health system by providing universal access to equitable, affordable and quality health care. The NRHM intended to reduce maternal and child mortality and OOPE on maternal care in rural areas of 18 states that had poor health infrastructure and health indicators. Subsequently, the program was extended to all the states and urban areas in the country and renamed as National Health Mission (NHM). The key components of NHM are *Janani Suraksha Yojana* (JSY) and *Janani Shishu Suraksha Karyakram* (JSSK). The JSY is a cash incentive scheme provided to mothers for delivering at public health centres or accredited health centers [18]. The incentive to mothers varies in rural and urban areas and in low and high performing states. Besides, it covers the incentive to Accredited Social Health Activist (ASHA). The JSSK, launched in 2011 entitled all pregnant women to deliver in public health institutions absolutely free and free treatment to sick infants up to 1 year. Over ten million pregnant women are provided cash incentives under the scheme annually. Details of the NHM are documented elsewhere [19].

**Fig. 1:**
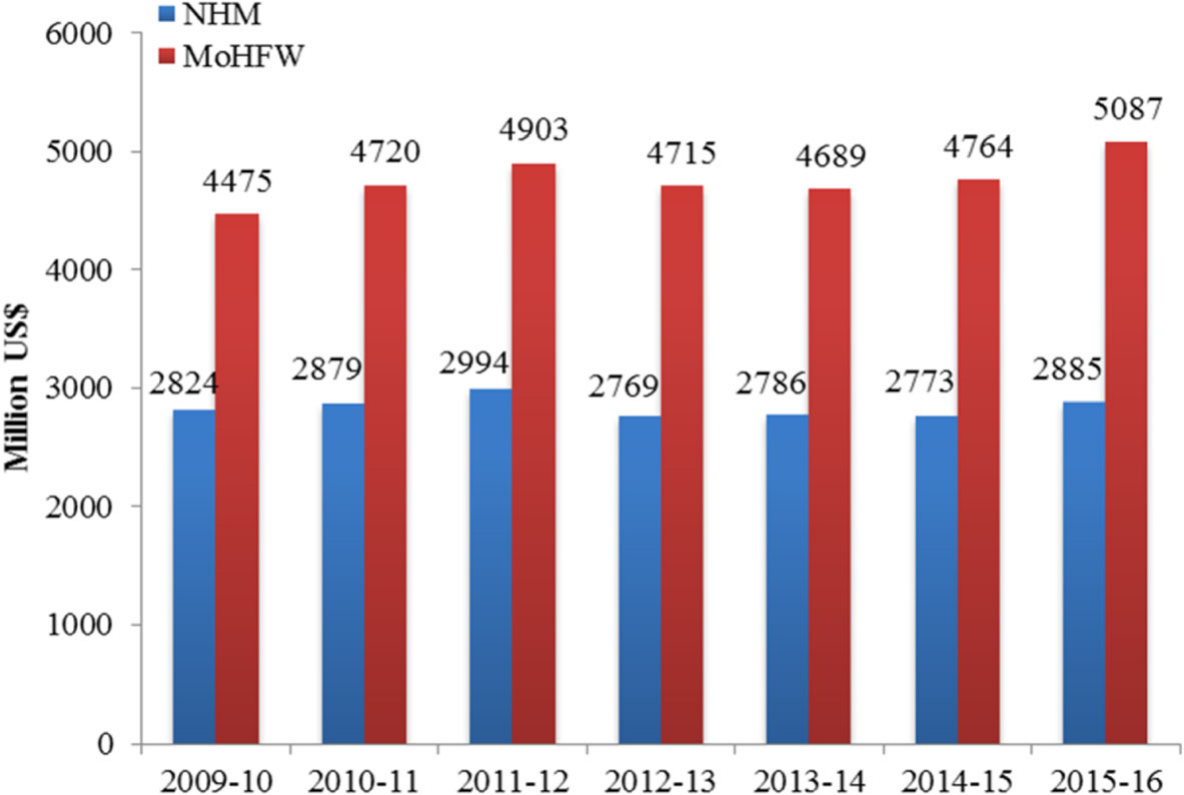
Trends in annual budget (US$) of Ministry of Health and Family Welfare (MoHFW), Govt. of India and National Health Mission (NHM), 2009–16 (at 2015–16 prices and 2015–16 exchange rate). Source: Kapur A, Srinivas V. Budget Brief 2015-16: National Health Mission Accountability Initiative [Internet]. New Delhi; 2016. Available from: http://www.cprindia.org/research/reports/budget-brief-2017-18-national-health-mission-nhm

Figure 1 presents the trends in health budget and that of NHM over time at constant prices [1US$ = 65.43]. The NHM accounts for more than half of the health spending of the central government over time. In 2014–15, about US$ 2626 million (Rupees 16,809 crores) were spent on NHM [20]. The recently released National Health Policy, 2017 highlights the success of NHM in the public health system and aim to reduce IMR to 26 per 1000 live births by 2019 and MMR to 100 per 100,000 live births by 2020 [21].

Since the implementation of NHM in 2005, there has been significant progress in maternal and child health in the country. Trends in IMR and MMR suggest substantial improvements in the post NHM period; IMR declined from 58 in 2004 to 40 per 1000 live births in 2013 (31% decline in the post NHM period) [22, 23] and MMR declined from 254 in 2004–06 to 167 per 100,000 live births in 2011–13 (34% decline) [24, 25]. Process indicators such as prenatal care, institutional delivery and postnatal care have shown significant progress in India [26]. JSY had a significant impact on increasing antenatal and natal care and reducing perinatal and neo-natal deaths [27]. Several small-scale studies have examined the success and constraints of the program [28–31]. Besides improving maternal and child health survival, the NHM was intended to reduce the OOPE and CHS on delivery, prenatal and post natal care. Though a number of studies have examined the differentials and determinants of maternal care [32–34], there are limited studies on the economic burden of maternal care over time in India.

The first systematic attempt on estimating OOPE and CHS on maternal care for India suggest high CHS to poor, rural households and the less educated [7]. The OOPE on delivery care varies largely across states and household characteristics [35]. Studies also found that maternal care in India placed a high economic burden on households and suggested reduction in OOPE to benefit the poor [36–38].

The aim of this paper is to estimate the OOPE and CHS on maternal care before the launch of the National Health Mission in India and a decade later. It addresses the research question whether the OOPE and CHS on maternal care have declined in the post NHM period? The paper has been conceptualized with the following rationale. First, large-scale investment in the public health programs of developing countries has large opportunity costs. Though there has been significant reduction in maternal and child mortality and increase in maternal care utilization in public health centres in the post NHM period, there is no study that has examined the OOPE by public and private health care providers in the pre and post NHM period. Understanding the level of OOPE in the pre and post NHM periods will help assess the functioning of the program and will be of immense help to multiple stakeholders. Second, both public and private entities in India are providers of maternal care services and are guided by varying principles. Hence, any systematic analyses in understanding the effectiveness of the program should focus on disaggregated analyses by type of provider (public-private). Third, high OOPE on health care is associated with higher CHS and increasing poverty [39, 40]. Studies also suggest that the medical care costs has been rising faster than the overall well-being of the households [41]. Reduction of CHS among the poor and marginalized was one of the main objectives of NHM and analyses on CHS need to be sensitive to account the health spending among the poor.

The paper is organized as follows: Section I gives a brief introduction of NHM and delineates the progress of maternal and child health in the post NHM period, Section II presents data and method, while Section III presents the results and Section IV provides discussion and conclusion.

## Methods

### Data

The unit data of the 25th Schedule of the 60th and 71st rounds of the National Sample Survey (henceforth referred to as 60th and 71st respectively) is primarily used in the analyses. Both rounds of the surveys are population based nationally representative surveys, similar with respect to design, content and coverage and provide comprehensive information on morbidity, health care and cost of hospitalization. The 60th round was held from January to June 2004 and the 71st round was held from January to June 2014. These time periods were best suited for analyses as 2004 was the pre NHM period and 2014 marked a decade since the implementation of the NHM (post NHM period). No health survey of NSS was conducted between 2004 and 2014. Information on natal care in 2014 was collected as part of hospitalization and missed the cost of deliveries conducted at home. We have created data at the women’s level to compute the OOPE and at the household level to compute CHS. In 2014 sample, there were 236 households that had more than one woman who had availed institutional delivery and there were 96 such households in 2004. On the other hand the CHS is used at household level, derived from household consumption expenditure, subsistence expenditure and household’s OOPE. The 60th round covered a total of 73,868 households and 383,338 individuals and the 71st round covered a total of 65,932 households and 333,104 individuals. The NSS uses a stratified multistage sampling design and samples are drawn from all states and union territories of India. Samples drawn are representative and provide robust estimates at the state level by rural and urban and selected characteristics. The sampling methodology and findings from these surveys are available in the respective reports [42, 43]. Since the expenditure on maternal care is analyzed for 2004 and 2014, we have adjusted the expenditure at 2014 prices for valid comparison using the price deflator for rural (agricultural labourer) and urban areas (industrial worker) and at 2001 base prices [44]. Estimates on OOPE is presented in US$ at the 2014 exchange rate. Besides, in calculating CHS, we have used the state specific poverty line (for subsistence expenditure) for rural and urban areas as recommended by the Planning Commission for 2004–05 and 2011–12 [45]. The poverty estimates are derived from the household consumption expenditure data based on calories intake. The per capita calories intake of 2400 for rural areas and 2100 for urban areas are demarcated as poverty cut-off point in India and make poverty estimates comparable across states of India. The corresponding money value is labeled as poverty line. The poverty estimates in India are usually provided at 5-year interval and the 60th and 71st rounds of NSS (health surveys) are close to that of time period that estimates poverty in India. Mother is the unit of analyses for estimating OOPE while household is the unit of analyses for CHS. The sampling weights are used in the analyses for representativeness of the sample.

### Outcome

OOPE and CHS are two outcome variables, computed for delivery care, and maternal care (pre-natal, natal and post-natal) by type of health care provider (public-private). In computing the maternal care from private and public sources, we consider only those women who availed these three services either from the public or private. The OOPE is defined as the expenditure incurred by the women during pre-natal, institutional delivery and post-natal care net of reimbursement. Expenditure on prenatal care, institutional delivery and postnatal care was directly available in the data set and were summed to obtain expenditure on total maternal care. The CHS is defined as the health spending over 40% of household’s capacity to pay. The consumption expenditure data of the household and the state specific poverty line is used in deriving the household’s capacity to pay.

### Covariates

Individual and household level covariates are used in the analyses. The household characteristics pertain to the head of the household while individual characteristics pertain to the woman. The selection of covariates is guided by availability of variables in data set and literatures. The covariates included are age, residence (rural/urban), educational level, caste^1^ (Scheduled Tribe, Scheduled Caste, Other Backward Classes and Others), religion (Hindu, Muslims and Others), monthly per capita consumption expenditure (MPCE) quintile and household type (labourer household and others). We have also introduced an interaction term in the regression model, namely, type of health care provider and time (pre and post NHM) to capture the effect of time and source of provider in explaining change in OOPE and CHS in India.

### Analytical methods

Descriptive statistics, estimation of CHS, log linear regression and the logit regression models are used in the analyses. In literature, two alternative approaches are used in estimating CHS, both using capacity to pay (CTP). The approach suggested by Berki (1986) and later by Van Doorslar et al. (2007) defines CHS as a proportion of consumption expenditure (usually 10% and more) [46, 47]. The limitation of this approach is its inability to account for the CHS of poorer sections as the poor spend less on health due to their lower ability to pay. Studies have demonstrated the limitation of this method in the empirical estimation of maternal expenditure [7]. The second approach by Xu et al. (2003) [3] derives CTP by deducting the subsistence expenditure (SE) and is largely used in literatures [48, 49]. It defines CHS;

1 $$ {\mathrm{CHS}}_{\mathrm{i}}={\mathrm{OOPE}}_{\mathrm{i}}/\Big(\mathrm{X}\mathrm{i}-\mathrm{f}\left(\mathrm{X},\Big)\right)>=\mathrm{z} $$

Where X_i_ is the consumption expenditure of i^th^ household and f(X) is the subsistence expenditure of the population. The SE is estimated either using median food expenditure or as poverty line of the specific country/region. Unfortunately, the health surveys in India do not collect detailed consumption expenditure and therefore the food expenditure is not available. In such cases, we have used the state specific poverty line to account for the subsistence expenditure. This approach is sensible where the poor are concerned, as those households below poverty line are classified as incurring CHS if they incurred OOPE for maternal care. The cut-off point of CHS is normative and usually taken as 40% in literature. Thus, a household is said to incur CHS if its health spending exceeds 40% of its capacity to pay.

To understand the impact of NHM on OOPE and CHS, we have pooled the variables of both rounds of health surveys. Two types of regression models are used; a log linear regression model for OOPE and a logit regression model for CHS. The log linear regression model was estimated as OOPE was continuous variable and skewed in nature. The logit model was used as the CHS was dichotomous variable, 0 for not incurring catastrophic health spending and 1 for incurring catastrophic health spending. Both set of models were estimated for delivery care and maternal care. We clustered standard error by first stage sampling unit (village/urban blocks). Each of the regression models were adjusted for state and time (survey-year) fixed effect. The fixed effects are captured at the state level because the states in India exhibit large variation in demographic, social, economic and health parameters. Also, health is a state subject and the state makes policies, programs and implement uniformly across the state. Besides, the state fixed effect model captures the unobserved factors in the regression model.

The regression models used for OOPE is defined as

2 $$ \ln\ \left({\mathrm{OOPE}}_{\mathrm{i}}\right)=\upalpha +{\upbeta}_1{\mathrm{res}}_{\mathrm{i}}+{\upbeta}_2{\mathrm{age}}_{\mathrm{i}}+{\upbeta}_3{\mathrm{e}\mathrm{duc}}_{\mathrm{i}}+{\upbeta}_4{\mathrm{mpceqt}}_{\mathrm{i}}+{\upbeta}_5{\mathrm{caste}}_{\mathrm{i}}+{\upbeta}_6{\mathrm{religion}}_{\mathrm{i}}+{\upbeta}_7{\mathrm{htype}}_{\mathrm{i}}+{\upbeta}_8\mathrm{INT}\_\mathrm{NHM}\_{\mathrm{SOU}}_{\mathrm{i}}+{\upbeta}_9{\mathrm{state}}_{\mathrm{i}}+{\mathrm{e}}_{\mathrm{i}} $$

where α is the intercept, res is residence (rural/urban), age is age of the woman, edu is educational level of the mother, mpceqt is monthly per capita consumption expenditure quintile of the household, caste is the caste of the household, religion is religion of the household, htype is the type of household (labourer or non-labourer household). INT_NHM_SOU is the interaction term computed based on time (pre / post NHM) and source of providers (public/private). These are public*preNHM, private*preNHM, public*postNHM and private*post NHM. The interaction term helps to understand the role of NHM in reducing the CHS in public/private health centres. A total of 36 states and union-territories are included in the analyses. The subscript i is used for ith woman.

Similarly, the regression models used for CHS is defined as

2 $$ \mathrm{logit}\ \left({\uppi}_{\mathrm{i}}\right)=\upalpha +{\upbeta}_1{\mathrm{res}}_{\mathrm{i}}+{\upbeta}_2{\mathrm{age}}_{\mathrm{i}}+{\upbeta}_3{\mathrm{educ}}_{\mathrm{i}}+{\upbeta}_4{\mathrm{mpceqt}}_{\mathrm{i}}+{\upbeta}_5{\mathrm{caste}}_{\mathrm{i}}+{\upbeta}_6{\mathrm{religion}}_{\mathrm{i}}+{\upbeta}_7{\mathrm{htype}}_{\mathrm{i}}+{\upbeta}_8\mathrm{INT}\_\mathrm{NHM}\_{\mathrm{SOU}}_{\mathrm{i}+}{\upbeta}_9{\mathrm{state}}_{\mathrm{i}} $$

where π_i_ is the probability of incurring catastrophic health spending for delivery/maternal care of ith household. The model estimates the log odds of incurring CHS adjusted for a set of explanatory variables. All explanatory variables are same as of Eq. (1) except age and education. Information on age and education of the head of the household is used, as household is the unit of analyses and there are cases where more than one woman had delivered from the same household. Results are presented with the help of regression coefficients, odds ratio and 95% CI.

## Results

The mean age of women was 25.52 years in 2004 and 25.69 years in 2014. In 2004, 52.03% women were illiterate, 43.48% had primary education and 4.49% had secondary and above education. In 2014, 28.58% women were illiterate, 23.24% had primary and 48.18% had secondary and above educated. The MPCE of the household was 1066 rupees in 2004 and 1311 rupees at 2014 prices. About 34.17% households were labourer households in 2004 compared to 28.46% in 2014 (Table not shown). Table 1 presents the definition of variables used and the descriptive statistics. The utilization of maternal care has increased from both public and private health providers and the increase was larger from public health center. On the other hand, the increase in OOPE in private health center is large compared to public health center. The standard deviation of OOPE on all three maternal care in private health center was 240 in 2004 and 455 in 2014 suggesting increasing variation over time. In case of public health center the standard deviation of all three maternal care had declined from 108 in 2004 to 86 in 2014 suggesting reduction in variability in OOPE. The standard deviation for institutional delivery was similar for OOPE.

**Table 1:** Definitions and descriptive statistics of variables on maternal care in India by source of provider, 2004–15

Variable	Definition	Descriptive statistics					
		Pre NHM (2004)			Post NHM (2014)		
		Public	Private	Total	Public	Private	Total
Ante-Natal Care (ANC)	Percentage of mothers who received any of the ante-natal check-ups during pregnancy.	43.34	32.04	75.38	55.13	35.30	90.43
Institutional Delivery	Percentage of mothers who delivered at either public or private health care facility	21.97	22.31	43.28	53.17	29.47	82.64
Post Natal Care (PNC)	Percentage of mothers who availed any post natal services following childbirth.	28.52	35.90	64.42	45.69	33.62	79.31
All three maternal care services	Percentage of mothers who received all of the three maternal care services (ANC, natal care and PNC).	11.32	11.54	31.80	31.22	20.20	66.09
Out-of-Pocket Expenditure (OOPE) on Institutional Delivery in US$	Total expenditure on institutional delivery net of reimbursement (mean)	42	170	56	46	300	138
Out-of-Pocket Expenditure (OOPE) on all three maternal care services in US$	Total expenditure on ANC, natal and post-natal care net of reimbursement (mean)	60	260	170	86	478	239

### Prenatal, natal and postnatal care in pre and post NHM periods

**Fig. 2:**
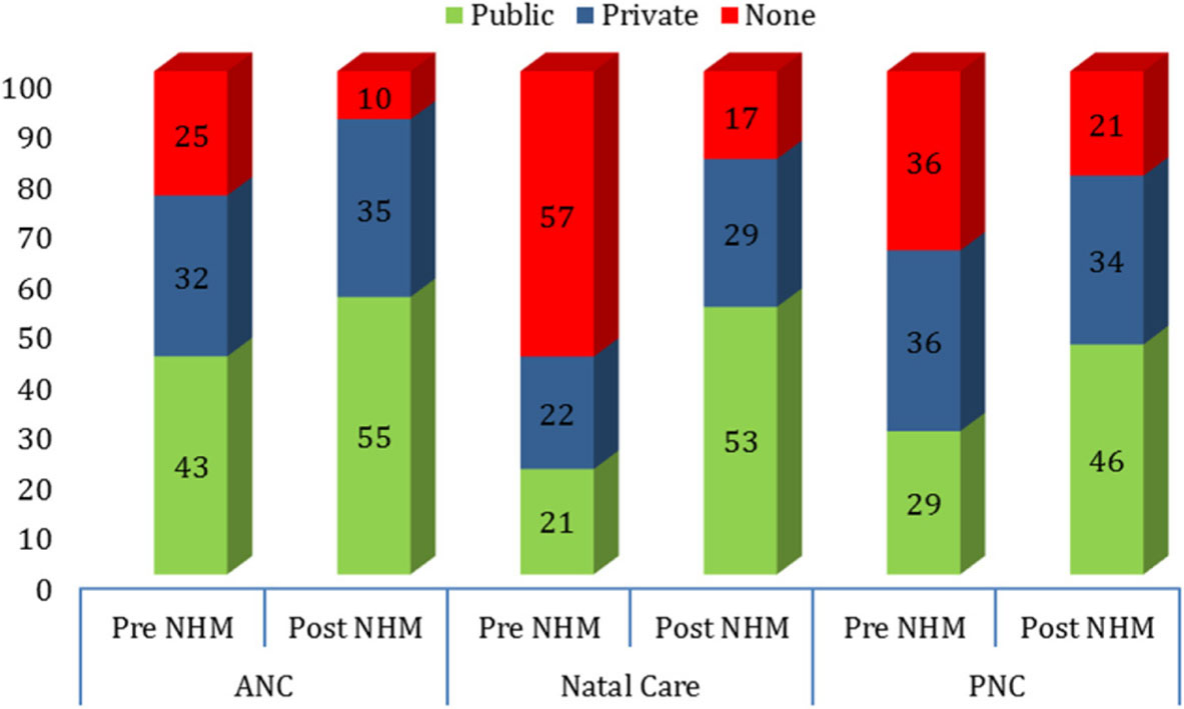
Percent distribution of women who received pre-natal, natal and post-natal care by public and private health centres during Pre and Post NHM Periods in India

**Fig. 3:**
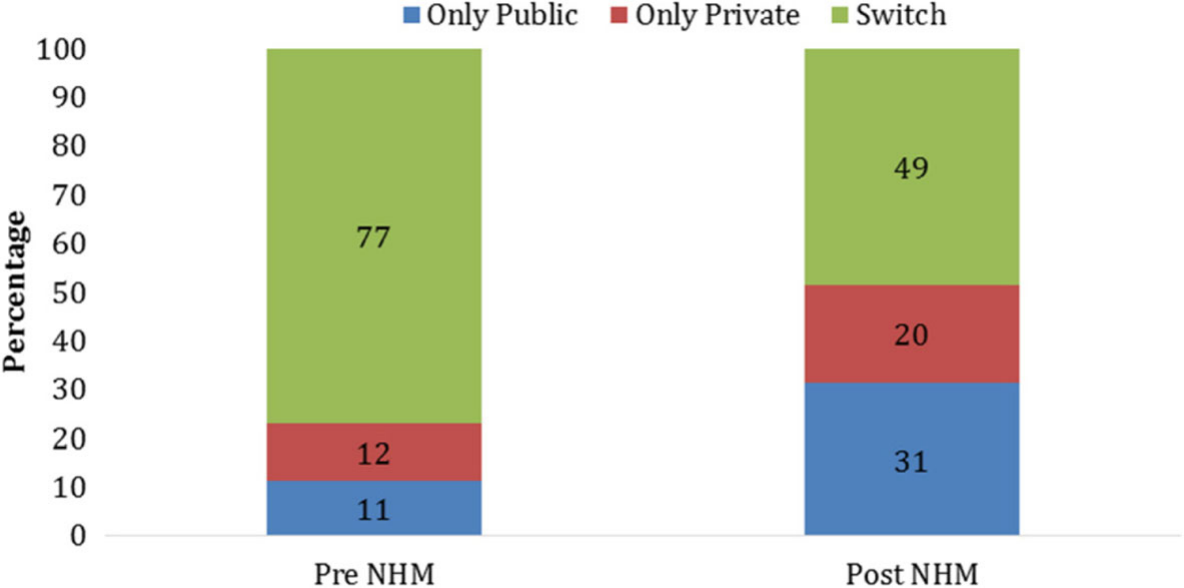
Percent distribution of women who availed prenatal, natal and postnatal care from public health care providers only, private health care Providers only and switch from public to private and vice versa in pre NHM and post NHM periods in India

Figure 2 presents the extent of prenatal, natal and postnatal care in the pre and post NHM periods by source of provider. The utilization of prenatal care had increased from 75% in 2004 to 90% in 2014. Postnatal care was estimated at 64% in 2004 and 79% in 2014. Among the three maternal care services, the increase in natal care was the highest while the postnatal care was the least during the post NHM period. Women who had availed all three services irrespective of the health care provider accounts 32% in 2004 and 66% in 2014. The pattern was similar for natal and post-natal care. Since 2004, all three maternal care has recorded increase in public health centres. For example, prenatal care from public health centres has increased from 43% in 2004 to 55% in 2014 while that in private health centres has increased from 32% to 35% during the same period. One interesting pattern that emerges is the continuity of maternal care services and decline in switching of services from public health centres in the post NHM period. Among those women who had availed all three services in 2004, 11% availed these services only from public health centres, 12% availed services only from private health centres and 77% switched from public to private or vice versa (Fig. 3). In 2014, among mothers who availed maternal care services, 31% availed only from public health centres, 20% availed only from private health centres and 49% switched services between private and public health centres.

### OOPE on prenatal, natal and postnatal care in pre and post NRHM periods

Appendix 1 presents the differentials in OOPE on institutional delivery (natal care) and all three maternal care services in 2004 and 2014 by type of health care providers. The OOPE on total maternal care is presented for those who availed all three services either from public or private health centres exclusively, and those who switched between public and private service providers during pregnancy and childbirth. Among women who availed all three services in public health centres, the mean OOPE had increased from US$60 to US $86 (by 43%) while that in private health centres has increased from US$260 to US$ 478 (by 84%). For those who switched services between private and public provider, the OOPE increased by 17%. The mean OOPE on all three services increased with the educational attainment of women and economic well-being of household, irrespective of type of health care providers. The OOPE on institutional delivery had increased from US$ 56 to US$ 138 during the pre and post NHM periods (at 2014 prices). The differentials in OOPE on delivery care in public health centres had increased marginally over time - US$ 42 in the pre NHM period and US$ 46 in the post-NHM period while that from private health centres had increased from US$ 170 in 2004 to US$ 300 in 2014 (by 76%). The differentials in OOPE in natal care in public health centres among women with no education or primary education had declined in the post-NHM period compared to the pre-NHM period, while it increased among those who had secondary, high school education and above. The differentials in OOPE from private health centres by MPCE quintile in the pre and post NRHM periods suggest that the OOPE among the poorest had increased threefold and that among the richest twofold.

Table 2 present result of log-linear regression model of OOPE for institutional delivery and maternal care. Women aged 25 years and above are likely to incur 5% more OOPE on delivery care and all three maternal care ((exp(0.05)-1)). The OOPE on institutional delivery and all three maternal care increases significantly with the economic well-being of the households. Compared to the poorest MPCE quintile, the OOPE on institutional delivery among the richest MPCE quintile was 55% higher (exp(0.44)-1)). The OOPE was higher among women with higher educational attainment and other than labourer households. The OOPE in post NHM period from public health center was 3% lower than that of pre NHM period (not statistically significant). The OOPE for institutional delivery from private health center in post NHM period was 5.62 times higher than that of public health center in pre NHM period. The OOPE for institutional delivery from private health center in post NHM period was even higher than that from private health center in pre NHM period. In post NHM period, the OOPE for all three maternal care from public health center had increased by 32% ((exp(0.28)-1) and statistically significant. In case of private health centers in post NHM period, the increase in OOPE on all three maternal care was about 5.62 times compared to that from public health center in pre NHM period. In general, the pattern on OOPE in maternal care is similar to that of institutional delivery.

**Table 2:** Regression coefficient and 95% confidence interval of OOPE on institutional delivery and total maternal care during pre and post NHM periods in India

Background characteristics	Institutional delivery		All three maternal care	
	Coef.	95% CI	Coef.	95% CI
Age of Mother				
< 25 (R)				
25+	0.05	0.02–0.08	0.05	0.01–0.08
Place of Residence				
Rural (R)				
Urban	−0.04	−0.08- -0.00	−0.01	−0.05-.0.03
Education Level				
No Education (R)				
Primary	0.07	0.02–0.12	0.14	0.08–0.21
Secondary	0.20	0.15–0.26	0.24	0.18–0.31
Higher secondary +	0.30	0.24–0.36	0.31	0.24–0.38
MPCE Quintile				
Poorest (R)				
Poorer	0.08	0.03–0.14	0.09	0.03–0.15
Middle	0.16	0.11–0.22	0.16	0.09–0.22
Higher	0.29	0.24–0.35	0.28	0.22–0.35
Highest	0.44	0.38–0.50	0.43	0.36–0.49
Caste				
Scheduled Tribe (R)				
Scheduled Caste	0.08	0.00–0.15	0.14	0.05–0.22
Other Backward Caste	0.13	0.06–0.20	0.20	0.12–0.28
Others	0.19	0.12–0.26	0.23	0.15–0.31
Religion				
Hindu (R)				
Muslim	−0.02	−0.07-0.03	−0.07	−0.12- -0.01
Others	0.02	−0.05-0.10	0.04	−0.04-0.11
Household Type				
Other (R)				
Labourer	0.08	0.04–0.12	0.07	0.03–0.12
Interaction effect				
Public*Time (PreNHM) (R)				
Private*Time (PreNHM)	1.43	1.35–1.51	1.52	1.41–1.62
Public*Time (PostNHM)	−0.03	−1.10-0.03	0.28	0.19–0.37
Private*Time (PostNHM)	1.89	1.82–1.96	2.02	1.92–2.11
Constant	7.60	7.43–7.76	8.08	7.91–8.25

(R): Reference Category

### Catastrophic health spending in pre and post NHM periods

Table 3 presents the differentials of CHS on maternal care and delivery care by public and private health centres and socio demographic characteristics in the pre and post NHM periods. Among mothers who availed all three maternal care from public health centres, the CHS has declined from 56% in the pre NHM period to 29% in the post NHM period and that from private health centres declined from 56% to 47% during the same period. The decline in CHS was quite substantial among all economic groups (except the poorest MPCE quintile) and all educational groups. The CHS on delivery care in public health centres had declined from 51% in 2004 to 20.5% in 2014 and that in private health centres has declined from 52% to 34% during the same period. Overall, the level of CHS on delivery care has declined from 52% in 2004 to 25% in 2014. The CHS on delivery care and maternal care reduced for many socio-demographic characteristics. The CHS on maternal care and delivery care is negatively associated with the economic status of the household, irrespective of the type of service and time.

**Table 3:** Percentage of households incurring catastrophic health spending on institutional delivery and all three maternal care by source of provider and selected characteristics during pre NHM (2004) and post NHM (2014) periods in India

Background characteristics	CHS on institutional delivery						CHS on all three maternal care			
	Public		Private		All		All three care from public health centers only		All three care from private health centers only	
	2004	2014	2004	2014	2004	2014	2004	2014	2004	2014
Age										
< 30	54.5	20.1	55.2	41.7	54.8	25.7	52.9	30.6	54.7	59.2
30–59	51.5	21.1	53.7	32.3	52.5	25.1	57.6	29.1	61.4	45.3
60+	45.4	19	45	33.6	45.3	25.1	56.8	28.3	45.4	43.2
Residence										
Rural	55.3	21.1	61.8	37.2	58.5	25.7	58.9	30.4	68.9	51.5
Urban	43.1	18	38.3	29.6	40.2	23.9	50.3	24.8	40.6	41.8
Education Level										
No Education	65.1	23.9	68.1	42.6	66.4	28.8	69	33.8	73.6	56.1
Primary	49.7	21.6	58.9	38.5	53.9	26.9	55.9	31.7	67.6	48.7
Secondary	31.8	18.1	36	33.2	34.4	23.8	48	24.7	32.6	48.8
High School+	25.5	8.9	32.8	20.6	30.6	15.5	15.8	16.4	38.8	34.6
Caste										
Scheduled Tribe	59.7	29.7	65.4	23.7	61.4	28.6	51.8	37.5	65.3	28.9
Scheduled Caste	59.4	22.2	63.8	48.4	61.1	28.3	59.1	33.3	68.4	56.8
Other Backward Caste	50.9	19.5	59.8	34.5	55.8	25.2	56.8	27.2	67.6	49.4
Others	42.5	15	38	29.5	39.9	21.6	51.8	24.1	38.9	43.2
Religion										
Hindu	52.1	20.5	51.7	34.8	51.9	25.4	55	29.8	55.6	48.3
Muslim	53.4	20.6	66.8	34.1	60.5	25.2	66.8	27.8	66.8	44.6
Others	37.4	20.4	28.8	26.3	32.6	23.1	52.7	26.8	33.3	39.1
Household Type										
Labourer	48.4	26	54.3	48.8	51.7	30.7	56.4	38.7	69.6	57.9
Others	51.9	18.4	51.5	32	51.7	20.6	55.2	25.7	52.4	46
MPCE Quintile										
Poorest	79.8	62.8	99	90.4	87.6	67.9	76.8	74.6	100	95.8
Poorer	79.3	7.4	95	57.3	87.3	22.3	80.4	23.5	99.4	68.4
Middle	68.3	2.3	87	29.4	75.8	11.2	69.5	6.3	95.5	51.8
Richer	31.6	0.9	48.2	19.2	40.1	8.6	40.7	3.2	61.2	37.8
Richest	8	0.9	19	8	15.6	5.1	17.8	0.4	25.3	20.7
State										
Non EAG	47.9	16.6	48.5	31.7	48.3	23.3	52.3	24.9	53.9	45.1
EAG	60.9	23.5	60.1	39	60.4	27.3	68.9	32.7	60.1	51.9
Total/India	51.4	20.5	52.1	34.2	51.7	25.2	56.2	29.4	55.6	47.2
Number of households	1835	8792	1866	5559	3701	14,351	1038	5263	910	4179

There were 38 households in 2014 and 8 households in 2004 where some women availed services in public and others in private health centres

Table 4 presents the odds of incurring CHS on all three maternal care services and institutional delivery. Age of the head of the household is a significant determinant of CHS for institutional delivery and all three maternal care. Urban households are less likely to incur CHS compared to rural households for institutional delivery (OR 0.29, 95% CI 0.26, 0.32) and all three maternal care services (OR 0.27, 95% CI 0.24, 0.31). The educational attainment of head of the household does not show consistent pattern for all three maternal care services. On the other hand, economic gradient is strong, negative and significant suggesting that CHS declines with economic well-being of the household. Type of households is not significant for maternal care services and institutional delivery. Maternal care (all three) in public health center in the post NHM period (OR 0.06, 95% CI 0.04, 0.07) are less probable to be catastrophic compared to public health center in pre NHM period. Similarly, all three maternal care services at private hospitals are significantly more likely to be catastrophic in the post NHM period compared to public health centres in the pre NHM period (OR 1.34, 95% CI 1.08, 1.67). In case of institutional delivery the odds of CHS from public (OR 0.03, 95% CI 0.02, 0.03) and private (OR 0.54, 95% CI 0.46, 0.63) health centers in post NHM period was significantly lower than public health centers in pre NHM period.

**Table 4:** Odds Ratio and 95% Confidence Interval (CI) of catastrophic spending on institutional delivery and total maternal care expenditure associated with socio-economic and demographic correlates during pre and post NHM periods in India

Background characteristics	Institutional delivery		All three maternal care	
	Odd ratio	95% CI	Odd ratio	95% CI
Age				
< 30 (R)				
30–59	0.86	0.77–0.97	0.78	0.68–0.89
60+	0.75	0.64–0.86	0.65	0.56–0.77
Place of Residence				
Rural (R)				
Urban	0.29	0.26–0.32	0.27	0.24–.0.31
Education Level				
No Education (R)				
Primary	0.98	0.87–1.10	1.02	0.89–1.18
Secondary	1.03	0.91–1.18	1.11	0.96–1.28
Higher secondary +	1.08	0.92–1.27	1.17	0.98–1.40
MPCE Quintile				
Poorest (R)				
Poorer	0.06	0.05–0.07	0.08	0.07–0.10
Middle	0.02	0.02–0.03	0.03	0.02–0.04
Higher	0.01	0.01–0.01	0.01	0.01–0.02
Highest	0.00	0.00–0.00	0.00	0.00–0.00
Caste				
Scheduled Tribe (R)				
Scheduled Caste	1.00	0.82–1.21	1.14	0.90–1.43
Other Backward Caste	1.18	0.99–1.42	1.44	1.16–1.78
Others	1.19	0.99–1.44	1.30	1.04–1.63
Religion				
Hindu (R)				
Muslim	0.88	0.77–1.01	0.77	0.65–0.91
Others	1.00	0.81–1.23	1.00	0.79–1.26
Household Type				
Other (R)				
Labourer	1.10	0.97–1.15	1.14	0.98–1.33
Interaction effect				
Public*Time (PreNHM) (R)				
Private*Time (PreNHM)	3.48	2.92–4.16	4.97	3.88–6.36
Public*Time (PostNHM)	0.03	0.02–0.03	0.06	0.04–0.07
Private*Time (PostNHM)	0.54	0.46–0.63	1.34	1.08–1.67
Constant	306.61	193.33–486.26	327.92	195.11–551.15

(R): Reference Category

## Discussion

The NHM in India was intended to increase the utilization of maternal care, reduce maternal mortality, neo-natal and infant mortality and reduce the OOPE and catastrophic spending on maternal care. Though evidence suggests an increase in the utilization of maternal care services and reduction in maternal and infant mortality in the post NHM period [50–52], there is no study that has examined the effectiveness of the program in reducing the OOPE on maternal care. Earlier studies were confined to a point of time or addressed particular services and none of these provided comparable estimates of OOPE in pre and post NHM by public and private health providers [7, 35, 38]. This study provides comprehensive estimates of OOPE and CHS in pre and post NHM periods in public and private health centres. The data set we have used is publicly available and the time period is best suited for analyses.

The salient findings are as follows. First, the OOPE on delivery care in public health centres (unadjusted mean at constant prices) has remained similar during the pre and post NHM periods and increased by 77% during post NHM period in private health centres. The OOPE on institutional delivery in private health centres was nearly four times higher than that in public health center in the pre-NHM period and increased by 6.5 times in the post NHM period. On controlling for socio-economic and demographic confounders and time, the OOPE on institutional delivery from public health center had not shown any significant increase in post NHM period. In case of private health centers, the increase in OOPE for institutional delivery was large and significant. This confirms that the overall increase in OOPE on institutional delivery was largely driven by increase in OOPE in private health centers. The OOPE on institutional delivery increased with educational attainment and MPCE quintile linking high OOPE to ability to pay and quality of care. Second, though, the OOPE on delivery care in public health centres has remained similar in pre and post NHM periods, it has increased significantly for all three maternal care services. The OOPE on all three maternal care in public health centers during post NHM period had increased by 32% (statistically significant) and that in private health centers had increased many fold. -Third, the CHS on delivery care and all maternal care services from public health centres in the post NHM period has declined by almost half. In the case of institutional deliveries, reduction of CHS in public health centres was experienced across all educational and economic classes. The decline in CHS was also noticeable in private health centres. With regard to all three maternal care services, the reduction of CHS was lower compared to that in delivery care but the level remained high. Fourth, the multivariate analyses confirmed decline in CHS in the post NHM period and a larger decline in the share of CHS in public health centres. Households residing in rural areas and with lower economic status are more likely to incur CHS and these findings are consistent with other studies [5, 6, 35, 38]. The interaction of time and NHM suggests that compared to those who delivered in public health centres during pre NHM period, those delivered or availed all there maternal care services in the post NHM period from public health centers were less likely to incur CHS in India. However, in case women availed all three maternal services from private health center in post NHM period, they are more likely to incur CHS.

These findings suggest that the NHM has been successful in increasing deliveries in public health centres and reduced the CHS in India. Increase in continuation of services in public health centres and reduction in switching from public to private healthcare providers is indicative of improvement in public health services on maternal care in India. The decline in OOPE from public health centres for the less educated and poor mothers may be attributed to the JSY under NHM. However, the use of pre-natal and post-natal has not recorded similar increase as that of institutional delivery in the post NHM period. This is possibly because the program priority under JSY was on increasing institutional delivery. The reduction of CHS is a reflection of the success of the program. Decline in CHS may be attributed both to the NHM and improvement in the economic well-being of the households. On the other hand, the differentials in OOPE among public and private health centres are large and there has been overall increase in OOPE on delivery care. This is possible because of increasing incidence of caesarian deliveries across the states of India.

We acknowledge some limitations of the study. First, a time series analyses could not be feasible due to data constraint. The NSS health survey was conducted in interval of 10 years; 2004 and 2014. No health survey of NSS was conducted in intervening period and so the time series analysis is ruled out. Similarly, the difference in difference analyses is not feasible as NHM was implemented in 18 poor performing states for initial few years and then expanded to advanced states. Getting control group may not feasible, as there were large differentials between poor performing and advanced states of India. Second, the analyses could not be performed separately for caesarian deliveries and normal delivery as the type of delivery (normal/caesarian) was not recorded in the survey. Third, the quality of maternal care that is often linked to cost were not collected in the survey and could not be analyzed. The indirect cost on hospitalization has not been estimated. Besides, improvement in health infrastructure in the post NHM period is beyond the scope of the study.

## Conclusion

Based on these findings, we conclude that the NHM is effective in increasing in utilization, continuation of services in public health centres and reducing OOPE and CHS in public health centres on maternal care. We suggest that the cash incentive under NHM should continue and private health care providers should be regulated with respect to pricing and quality of care. The program should focus on improving the quality of services in public health centres. Besides, we recommended that the forthcoming health survey (NSS) should integrate an abridged version of the consumption schedule, question on expenditure on home delivery and a separate code for caesarian and normal delivery is recommended.

## Appendix

**Table 5:** Out-of-pocket expenditure (in US$ at 2014 prices) on institutional delivery and maternal care (pre-natal, natal and postnatal) by public and private health centres and selected characteristics during pre NHM (2004) and post NHM (2014) periods in India

Background	OOPE on institutional delivery						OOPE on all three maternal care services					
	Public		Private		All		All three care from public only		All three care from private only		Switch from public to private and vice versa	
	2004	2014	2004	2014	2004	2014	2004	2014	2004	2014	2004	2014
Age												
< 25	39	47	166	279	57	128	51	88	271	443	194	207
25+	45	46	175	319	56	146	70	84	248	507	195	248
Residence												
Rural	44	44	155	274	44	113	60	84	230	451	201	214
Urban	36	54	191	340	100	206	58	95	294	513	183	264
Education												
No Education	36	33	104	229	27	75	49	65	156	370	137	153
Primary	44	42	172	233	75	90	66	79	271	391	193	184
Secondary	47	53	277	278	214	131	52	101	340	431	409	227
High School+	57	70	236	371	203	263	111	109	316	570	405	324
Caste												
Scheduled Tribe	28	37	130	193	24	65	53	67	145	300	255	191
Scheduled Caste	33	41	137	299	37	106	45	78	245	467	144	196
OBC	42	44	155	297	55	143	63	82	269	473	162	231
Others	53	61	201	322	87	181	72	116	266	515	254	248
Religion												
Hindu	41	44	168	302	56	136	55	83	259	479	198	227
Muslim	32	56	167	268	49	131	52	97	263	438	144	213
Others	68	57	195	381	97	181	142	94	266	590	265	271
Household Type												
Labourer	32	41	129	256	36	90	49	77	223	418	159	199
Other	47	49	182	309	67	155	66	90	268	488	207	237
MPCE Quintile												
Poorest	30	36	88	246	24	75	34	70	116	406	99	176
Poorer	36	41	123	253	34	94	56	78	168	384	165	195
Middle	44	51	118	253	45	123	60	99	214	393	144	216
Richer	41	54	195	282	82	159	62	102	302	445	167	241
Richest	75	77	234	405	167	295	122	107	321	625	362	370
State												
Non EAG	35	52	179	324	76	179	48	90	281	497	186	233
EAG	59	42	149	256	39	96	89	83	205	432	215	216
India	42	46	170	300	56	138	60	86	260	478	194	226
Number of women	1874	8931	1923	5656	3797	14,587	1059	5513	944	4054	811	2428

## Abbreviations

ASHA: Accredited social health activist
CHS: Catastrophic health spending
CTP: Capacity to pay
EAG: Empowered action group
JSSK: *Janani Shishu Suraksha Karyakram*
JSY: *Janani Suraksha Yojana*
MPCE: Monthly per-capita consumption expenditure
NHM: National health mission
NRHM: National rural health mission
NSS: National sample survey
OOPE: Out-of pocket expenditure
Pl: Poverty line
SDG: Sustainable development goals

## Footnotes

1 The population of India are generally categorized into four caste groups: Scheduled Tribe (ST); Scheduled Caste (SC); Other Backward Caste (OBC) and others. Conventionally, the ST population are socially and economically the poorest section of the population followed by SC and OBC. Based on this classification, the central. State and local government provides reservation on education, employment and other benefits to ST, SC and OBC population.
